# Satisfaction with Daily Occupations for Elderly People (SDO-E)—Adaptation and Psychometric Testing

**DOI:** 10.3390/healthcare5040061

**Published:** 2017-09-25

**Authors:** Jenny Hultqvist, Birgitta Wästberg, Mona Eklund

**Affiliations:** 1 Department of Health Sciences, Faculty of Medicine, Lund University, Lund SE-221 00, Sweden; mona.eklund@med.lu.se; 2 Skane University Hospital, Malmo-Lund SE-221 85, Sweden; birgitta.wastberg@skane.se

**Keywords:** activity level, healthy ageing, occupational therapy, self-rated health

## Abstract

Satisfaction with everyday occupations has been shown to be important for health and well-being in various populations. Research into satisfaction with everyday occupations among elderly persons is, however, lacking. The aim was to investigate the psychometric properties of an adapted test version of the Satisfaction with Daily Occupations instrument (SDO) for elderly people, called SDO-E. Five hospital-based occupational therapists working with elderly people evaluated the content validity and usability of the SDO-E. The elderly participants consisted of 50 people from outside of the health services and 42 inpatients at an internal medicine clinic. They completed the SDO-E and rated their perceived health, activity level, and general satisfaction with daily occupations. The SDO-E showed fair content validity and utility, acceptable internal consistency, good preliminary construct validity and relevant known-groups validity. The SDO-E thus appears to be a useful screening tool for assessing activity level and satisfaction with daily occupations among elderly people, and a complement to other self-report instruments concerning factors connected with health and well-being. Future research should further explore the content validity of the SDO-E, particularly the views of the elderly themselves, and investigate the SDO-E in terms of sensitivity to change.

## 1. Introduction

Social and medical developments, including improved economic conditions and better healthcare, have led to increased longevity, and given the elderly population greater possibilities for retaining their health and well-being over a longer period of time [1]. Research shows that there is a positive association between engagement in everyday occupations and health and well-being in older adults [2,3,4]. There is an interest in healthy ageing internationally, which has been described as a multidimensional concept including both the absence of disease and disability, the maintenance of physical and cognitive abilities, and the commitment to social and productive activities [5]. Research in this field is not only based on models with a biological perspective focusing on disease and disability, but also on psychosocial models that highlight health factors associated with ageing. Successful ageing is described in the latter as an active life with everyday occupations, personal development, a perception of health and well-being and meaning and purpose in life [6]. The concept of “everyday occupations” encompasses all meaningful activities an individual may engage in over the course of a day. A person retiring may experience satisfaction and a sense of well-being, moving away from a demanding and/or stressful work situation [7]. Conversely, the retirement process may lead to a diminished sense of well-being, as the individual loses his/her occupation, which is related to paid work and a social network of co-workers [7]. Research has shown that replacing work with everyday occupations perceived as meaningful is critical for the experience of a good life as a retiree [8].

A subjective experience of occupation, such as satisfaction with everyday occupations, has been shown to be important for well-being in various populations [9,10,11]. However, a similar research focus on satisfaction with everyday occupations, but aimed specifically at elderly people, appears to have been neglected. This may be due to a lack of instruments for assessing satisfaction with everyday occupations in this group, and the aim of the current study was thus to contribute to filling this gap.

Satisfaction with everyday occupations has been defined in several different ways. Wilcock [12] highlights three perspectives on satisfaction in relation to occupation: (1) occupations necessary for our survival; (2) occupations that enable us to use and develop our skills; and (3) occupations that afford personal development. Morgan [13] argued that occupational satisfaction is also dependent on the value that the person attributes to the activity. Subjective experiences of occupation, such as satisfaction with everyday occupations, have been shown to be more related to positive health and well-being than to the actual doing [14,15]. Moreover, there are several studies that emphasize that subjective experiences of occupations are of importance for successful ageing [16,17], while on the other hand, there is little research that focuses on the experiences of satisfaction with everyday occupations among elderly people.

The Satisfaction with Daily Occupations (SDO) instrument [18] is one of a few that targets satisfaction with everyday occupations. The instrument includes the categories of work, leisure, domestic activities and personal care. It has been used to explore satisfaction among individuals of working age, such as people with psychiatric disabilities [19], with stress-related ill health [20], with rheumatic disease [10], and with a foreign background [21,22]. In summary, there appears to be a need for an adapted version of the SDO that explores satisfaction with daily occupations in a broader sense that has less focus on competitive work in order to better suit the situation of elderly people. The aim of the study was to develop and test an adapted pilot version of the SDO for elderly persons (≥70 years). The psychometric properties to be tested were internal consistency, content validity, usability, known-groups validity, preliminary construct validity, and floor and ceiling effects.

## 2. Materials and Methods

The present pilot study had a quantitative, cross-sectional design. The study was conducted in two stages: (1) Developing an adapted test version of the SDO, based on a 13-item version [20]; and (2) Collecting data to evaluate the validity, including content validity and the usability of the pilot version of the SDO for elderly people, hereafter termed SDO-E.

### 2.1. Development of a Test Version of the SDO-E

The original 9-item SDO [18] for use in a mental health context was expanded into a 13-item version [20] for use in a primary care context. This latter version served as the starting point for developing the SDO-E. The SDO is interview-based, and consists of four categories of items: work, leisure, domestic activities and personal care. Each category has 3–4 items where the person first answers whether he/she currently performs the activity or not. The sum of yes-answers forms a measure of the level of activity. After answering yes or no, the person rates his/her level of satisfaction with the activity. This is done whether the person currently performs the activity or not and the satisfaction is rated on a scale from 1 = least possible satisfaction to 7 = best possible satisfaction. The SDO-13 contains three questions regarding work. These focus on being presently employed, having worked within the past two months and participating in vocational rehabilitation. In order to better suit the situation of people of retirement age, these three questions were replaced by two other questions, one asking whether the person is presently working (paid or unpaid) and the other whether the person is presently attending any type of day center offering work-like activities. The remaining items from the SDO-13 were retained, and the pilot version of SDO-E thus consisted of 12 items, with a possible scoring of 0–12 on activity level and 12–84 on activity satisfaction. The 12 items are presented in Table 1.

### 2.2. Psychometric Testing of the SDO-E

#### 2.2.1. Study Context and Participants

The study was performed in two separate contexts: within and outside of the healthcare services. Senior citizens’ organizations and municipal day centers in the south of Sweden were approached for recruiting elderly people from outside of the health services. An internal medicine inpatient setting located in the south of Sweden was selected from within the health services, where elderly people with a known diagnosis were sought. The study participants consisted mainly of elderly people, who responded to the pilot version of SDO-E, but also occupational therapists (OTs) in the inpatient context who administered the pilot version of the SDO-E in that context and completed a questionnaire about content validity and usability.

#### 2.2.2. The Elderly Participants

The elderly participants in both contexts were recruited through convenience sampling. The inclusion criterion for the participants in both contexts was an age of ≥70 years, with the exclusion criteria of a diagnosis of dementia or learning disability. The occupational therapists assessed this for the inpatient sample, based on the information available in the medical records. For the other sample, the day centers that were approached did not admit people with dementia or learning disabilities. There are specific day centers for these groups in Sweden, which is why screening for cognitive impairments was not applied.

The aim was to attain a sample of 50 people from each context. This figure was arrived at after a general power calculation, in which it was assumed that the SDO-E could detect an effect size of 0.6 at *p* < 0.05 with 80% power if 85 participants were included. We had to rely on a general appreciation of effect size, given that the SDO had not previously been used with elderly people. Seeking 50 participants from each group was a strategy based on the likelihood that a few would decline to take part. The total number of elderly participants was 92.

Two undergraduate OT students assisted the researchers in recruiting 50 participants from the senior citizens’ organizations and municipal day centers for seniors, in both rural and urban areas. All the members of the addressed organizations were invited to participate in the study. New organizations with prospective participants were included until the goal of 50 participants was reached. These were recruited from six different settings, four citizens’ organizations and two day centers.

Eleven OTs working at either an orthopedic- or internal medicine clinic were invited to the study. Six OTs at the internal medicine clinic consented to participate and thus administered the SDO-E. They were asked to consecutively recruit 10 patients each, since it was expected that some would decline participation in the study. The OTs recruited between 2 and 10 participants each, resulting in 42 from the inpatient setting.

#### 2.2.3. The Participating Occupational Therapists

The six OTs working in inpatient internal medical care at a hospital in the south of Sweden administered the pilot version of the SDO-E in that context. They were all women with an average age of 28.3 years, ranging between 24 and 37 years. They had been working as OTs for 1–10 years, with an average of 4.7 years. They were also asked to complete a questionnaire about their perception of the content, usability, and time needed for administering the pilot version of the SDO-E. One of the participating OTs left for parental leave at the time and thus did not complete this questionnaire.

### 2.3. Data Collection

The data were collected by means of the pilot version of the SDO-E, and a questionnaire focusing on socio-demographics was completed by the elderly participants. The data collection took place in a secluded room in all settings and took approximately 30 min. The OTs working in the inpatient setting completed a questionnaire addressing content validity and usability. The data in the inpatient group were collected from December 2013 to April 2014, and the data for the group outside the health services were collected during the spring of 2015.

#### 2.3.1. Socio-Demographic and Clinical Data

A questionnaire for socio-demographic and clinical data was devised specifically for the present study. The participants answered questions about gender, age, civil status, educational level, and living situation. For the people with a known diagnosis, there were two additional questions concerning diagnosis and reason for hospitalization. This information was collected by the occupational therapists based on information from the patients’ medical records.

#### 2.3.2. Questions to Evaluate Construct Validity

The elderly participants in both groups answered two questions for the evaluation of the preliminary construct validity of the SDO-E. The first concerned satisfaction with everyday occupations in general, and was rated on a five-point rating scale where 1 = greatest satisfaction. A five-point scale was used to further distinguish this question on overall satisfaction with everyday occupations from the SDO-E items. The second was a question concerning self-rated health from the MOS short form-36 (SF-36) [23]. The participants were asked “how would you say your health is in general”, rated on a five-point rating scale where 1 = best possible health. This single item measure has been found to be a valid broad summary rating of health [23].

#### 2.3.3. Questionnaire for Evaluating Content Validity and Usability

The questionnaire used in the present study has been used previously and has been found to provide valuable information about an instrument´s content and usability [24]. The questions covered the following areas:

1. How easily could the content of the items be understood?
2. How well did the questions cover ‘satisfaction with daily occupations’?
3. Was there anything missing in the SDO-E concerning ‘satisfaction with daily occupations’?
4. If so, what was missing?
5. Are there questions that do not concern ‘satisfaction with daily occupations’?
6. If so, which questions?
7. How much time was needed to complete the SDO-E?
8. How reasonable was the time needed to complete the SDO-E?

Items 1, 2 and 8 were answered in accordance with an ordinal scale ranging from 1 to 5, where 1 denoted the most positive category, that is, ‘Very easily’, ‘Very well’ and ‘Very reasonable’, respectively. The most negative category (=5) was worded ‘Not easily at all’, ‘Not well at all’, and ‘Not reasonable at all’, respectively. Items 3 and 5 were answered with a yes or no, while item 7 asked for the minimum and maximum amounts of time used with clients when completing the SDO-E. Finally, items 4 and 6 were open-ended questions.

#### 2.3.4. Ethical Considerations

In accordance with the Act (2003:460) concerning the Ethical Review of Research Involving Humans [25], the participants gave written informed consent regarding the collected data being used in a study on psychometric properties. This concerned both those without a known diagnosis and the inpatient participants. The study was conducted in accordance with the Swedish law on research ethics [25] and was approved by the Regional Ethical Review Board in Lund, reg. no. 2017/453.

### 2.4. Data Analyses

Internal consistency was assessed by Cronbach’s alpha and, in accordance with Streiner and Norman [26], an alpha value between 0.70 and 0.90 and an item-total correlation between 0.20 and 0.80 were considered acceptable cut-offs. Non-parametric tests were used for further statistical analyses since the data were mainly based on ordinal scales. The psychometric analyses and methods of this study are presented in Table 2. In terms of the construct validity, it was hypothesized that the SDO-E activity level and the satisfaction scale would show a moderate to strong correlation with overall satisfaction with everyday occupations and with self-rated health. The limits for strength of correlations were set in accordance with Cohen [27]; <0.3 was seen as weak correlation, 0.3–0.5 as moderate and >0.5 as strong. The data analyses were performed with the IBM-SPSS software, version 23 (Chicago, IL, USA). The level of significance was set at <0.05.

## 3. Results

The characteristics of the elderly participants are presented in Table 3, and of those in the group with a known diagnosis a majority (69%) had a pulmonary illness and/or heart failure, with patients being treated for cancer and pneumonia also taking part (Table 3).

### 3.1. Internal Consistency

The Cronbach’s alpha for the present sample was 0.83. The corrected item total correlation ranged between 0.394 and 0.675 with a mean of 0.472. It should be noted that the test of internal consistency only concerned the satisfaction scale, and not the activity scale.

### 3.2. Content Validity and Usability

All responses about content validity and usability are summarized in Table 4. The question focusing on how well the SDO-E covered the construct of ‘satisfaction with daily occupations’ rendered a median rating of three (medium well). Two OTs gave a rating of two (fairly well), two gave a rating of three (medium well) and one OT gave the rating four (not so well). Most of the OTs did not think that anything was missing in the SDO-E concerning ‘satisfaction with daily occupations’. The one comment on what was missing was “some of the questions were not relevant”. Three OTs answered yes to the question whether there were items in the SDO-E that did not concern ‘satisfaction with daily occupations’. Two of these OTs commented on the questions about employment as being redundant due to their patients being retired. One OT considered cultural activities to be redundant for the elderly patients due to their multiple health problems.

Regarding usability in terms of how easy it was to understand the content of the items, the median rating was two (fairly easy). Concerning the time taken to respond to the SDO-E, the OTs found the time reasonable, the median rating being ‘fairly reasonable’.

### 3.3. Known-Groups Validity

For the group from outside of the health services, the activity level median was 8, with a range between 4 and 12, while the inpatient participants’ activity level median was 5, ranging between 1 and 8. For the summed satisfaction with daily occupations, the median (range) was 77 (57–84) for the group from outside of the health services, and 70 (21–84) for the inpatient participants.

The discriminating ability of the SDO-E was evaluated by comparing the two samples of elderly people. The analysis regarding activity level showed a significant difference between the groups (chi2 = 54.17; df = 1; *p* < 0.001), those from outside of the health services scoring higher. Similarly, the result of the analysis comparing the total satisfaction with daily occupations score showed that the group from outside of the health services scored significantly higher (*p* = 0.003) than the inpatient group.

### 3.4. Construct Validity

The SDO-E scale denoting activity level showed a moderate correlation with overall satisfaction with daily occupations (r_s_ = 0.426; *p* ≤ 0.001) and a strong association with self-rated health (r_s_ = −0.530; *p* < 0.001). For the summed satisfaction with daily occupations the analyses showed a moderate association with overall satisfaction (r_s_ = 0.460; *p* < 0.001) and a strong correlation with self-rated health (r_s_ = 0.522; *p* < 0.001).

### 3.5. Floor- and Ceiling Effects

The analysis of the distribution of frequencies regarding both activity level and summed satisfaction with daily occupations showed that less than 15% of the respondents achieved the lowest or highest scores, respectively, thus indicating an absence of both floor and ceiling effects.

## 4. Discussion

This study investigated the psychometric properties of the SDO-E, an adapted version of the SDO aimed for use with elderly people. The SDO-E satisfaction scale showed an acceptable internal consistency, as indicated by a Cronbach’s alpha higher than 0.80 and all corrected item total correlations being >0.30 [28], which is in line with the psychometric properties found in previous studies of the internal consistency of the SDO. The activity level score is only based on the number of activities the person is engaged in and calculating internal consistency is therefore not relevant.

To evaluate the content validity of the SDO-E, five OTs in an inpatient setting responded to a question about how well the SDO-E covered the construct of ‘satisfaction with daily occupations’, rendering a rating of medium well. Furthermore, the OTs were asked if anything was missing or redundant in terms of the construct of ‘satisfaction with daily occupations’. Two OTs commented that questions about employment were not relevant since their patients were retired, and one OT considered cultural activities (e.g., going to the theatre or attending concerts) as redundant due to the patients being elderly with multiple health problems. This result is not surprising when considering that the OTs’ responses were most likely based on their specific clinical expertise of a vulnerable group of elderly people. It should be noted, however, that although these participants were engaged in fewer activities compared to the participants outside of the health services, they were still very satisfied with their everyday occupations (as indicated by a median satisfaction score of 83% of the maximum score), although many of them were not currently involved in all the activities of the SDO-E. That a high level of satisfaction is perceived regardless of whether a person is currently engaged in the activity or not is in line with the intentions behind the SDO, and has also been demonstrated in previous research [11]. In order to obtain as broad a picture of satisfaction with everyday occupations as possible, which seems essential when considering the importance of satisfaction with everyday occupations for health and wellbeing, we thus maintain that it is important to retain all the items in the SDO-E. Further research focusing on exploring the content validity of the SDO-E for elderly people, and in particular the views of the elderly themselves appears, however, to be warranted. In terms of the usability, the OTs found the items of the SDO-E fairly easy to understand, and reported that the time needed to complete the instrument was reasonable, thus generating some support for the clinical usability of the SDO-E.

Furthermore, the results showed that the SDO-E had the ability to discriminate between elderly people outside of the health services and those with multiple health problems, thus demonstrating known-groups validity. As expected, participants outside of the health services reported significantly higher activity levels and satisfaction scores than the inpatient group. These findings are in line with previous research on the SDO’s discriminating ability when comparing a healthy sample with a sample with illness/disability [29].

The SDO-E scale denoting activity level showed, as hypothesized, moderate to strong correlations with overall satisfaction with daily occupations and self-rated health. Furthermore, the summed satisfaction with daily occupations showed a moderate to strong association with overall satisfaction and self-rated health. We thus propose that the SDO-E displays good initial construct validity. The results are interesting when considering the importance of occupational engagement, perceptions of everyday occupations and subjective perceptions of health and well-being for healthy ageing [17,30] and, in particular, the consequential need for relevant measures in these respects. The SDO-E may thus be a complement to other self-report instruments when aiming to assess factors connected with healthy ageing.

Finally, the frequency distribution showed that there were no floor or ceiling effects concerning activity levels or satisfaction scores [28].

### Limitations

Five OTs working in inpatient care reviewed the content validity of the SDO-E. Their perspective, although important, is likely to be limited to their area of clinical expertise, and as such may differ from a perspective on elderly persons in general. Furthermore, this study was not based on systematic sampling. Convenience sampling, which is common in pilot studies such as the current one, limits the generalizability of the findings. One could also question why we did not request information about health conditions in the group selected outside of the health services and about more socio-demographic data in general. This would have permitted a number of additional analyses in terms of group comparisons and factors associated with satisfaction with everyday occupations to be made. This study was the first to investigate a pilot version of SDO-E, however, and the aim was restricted to studying initial psychometric properties. Older people’s satisfaction with everyday occupations and associated factors will be a topic for future studies once we have established that the SDO-E has acceptable initial psychometric properties. Finally, in the assessment of construct validity, only two items (overall occupational satisfaction and general health) were used, which may be regarded a weakness. There is, however, research that strongly supports the use of single-item questions [23,31]. Given these limitations, we regard the findings concerning the psychometric properties of SDO-E as promising but preliminary.

## 5. Conclusions

The SDO-E showed acceptable internal consistency, good preliminary construct validity and relevant known-groups validity. We propose that the SDO-E shows promise as a useful screening tool for assessing activity level and satisfaction with daily occupations among elderly people, and that it may be a complement to other self-report instruments concerning factors connected with healthy ageing. Further research focusing on exploring the content validity of the SDO-E, in particular from the view of the elderly themselves, appears, however, to be warranted. Future studies should also investigate the SDO-E in terms of sensitivity to change, which can be of importance for use in outcome research.

## Figures and Tables

**Table 1:** Item content of the 12-item test version of the SDO-E.

Item content of the 12-item test version of the SDO-E
1 Employed at least two hours/week in the past two months
2 Attending day center in the past two months
3 Engaged in organized leisure occupations/hobbies at least once a week in past two months
4 Performing leisure occupations/hobbies on one’s own at least once a week in past two months
5 Taking part in cultural occupations at least once a week in past two months
6 Doing household work, such as cleaning and cooking, almost daily in past two months
7 Doing repairs and/or gardening in past two months
8 Organizing and planning the household work in the past two months
9 Taking care of children, parent or other close persons at least once a week in the past two months
10 Managing own personal hygiene on a daily basis
11 Doing physical exercises at least once a week in the past two months
12 Doing activities to relax at least once a week in the past two months

**Table 2:** Psychometric analyses and methods.

Psychometrics	Data	Methods of Analyses
Internal consistency	The satisfaction scale of the SDO-E	Cronbach’s alpha and corrected item-total correlations
Content validity	Questions 1–6 in the questionnaire for evaluating content validity and usability	Quantitative and qualitative description
Usability	Questions 7–8 in the questionnaire for evaluating content validity and usability	Quantitative description
Known-groups validity	The satisfaction scale and the level of activity scale of the SDO-E	Inferential statics, comparisons between the two groups. The Chi-Square Test and the Mann-Whitney U-Test
Construct validity	The satisfaction scale of the SDO-E, the overall level of activity item and the item addressing self-rated health	Correlations between the SDO-E scales and ratings of overall satisfaction with everyday occupations and self-rated health. Spearman’s Rank Correlation
Floor- and ceiling effect	The satisfaction scale and the level of activity scale of the SDO-E	Descriptive statistics, distribution of frequencies

**Table 3:** Socio-demographics of the elderly participants.

Variable	Group Outside Health Services (*n* = 50)	Group in Inpatient Care (*n* = 42)
**Gender**: male/female, n(%)	19(38%)/31(62%)	12(29%)/30(71%)
**Age**: mean(min–max) (SD)	77.3(70–88) (5.0)	81.6(70–97) (7.1)
**Civil status**: Single, n(%) Married/cohabitant, n(%)	21(42%) 29(58%)	30(71%) 12(29%)
**Living conditions**: House, n(%) Condominium, n(%) Rented apartment, n(%) Other, n(%)	13(26%) 20(40%) 13(26%) 4(8%)	6(14%) 33(79%) 1(2%) 2(5%)
**Educational level**: Elementary school or lower, n(%) Secondary school, n(%) University degree, n(%) Other, n(%)	32(64%) 6(12%) 8(16%) 4(8%)	26(62%) 7(17%) 9(21%) 0
**Diagnosis**: Chronic obstructive pulmonary disease or dyspnea, n(%) Heart disease or heart failure, n(%) Cancer, n(%) Pneumonia, n(%) Other (e.g., severe headache, osteoporosis, general health decline), n(%)		20(48%) 9(21%) 3(7%) 3(7%) 7(17%)

**Table 4:** Response distribution for the questionnaire about content validity and clinical utility.

Questions	Content Validity	Response Category/Number of Responses					
How well did the questions cover ‘satisfaction with daily occupations’?	1 = Very well; 5 = Not well at all	1/0	2/2	3/2		4/1	5/0
Was there anything missing in the SDO-E concerning ‘satisfaction with daily occupations’?	No/Yes	No/4			Yes/1		
If so what was missing?	Open-ended	Some questions were not relevant; patients are elderly with multiple health problems.					
Are there questions not concerning ‘satisfaction with daily occupations’?	No/Yes	No/2			Yes/3		
In this case which questions?	Open-ended	Item 1 employment and item 5 taking part in cultural activities *					
	**Usability**						
How easily could the content of the items be understood?	1 = Very easily; 5 = Not easily at all	1/0	2/3	3/3		4/0	5/0
How much time was needed to complete the SDO-E (in minutes)?	Least, mdn(min–max) Most, mdn(min–max)	11.6(8–15) 18.4(12–25)					
How reasonable was the time needed to respond to the SDO-E?	1 = Very reasonable; 5 = Not reasonable at all	1/2	2/2	3/1		4/0	5/0

* E.g., going to the theatre or attending concerts.
